# 350 nm Broadband Supercontinuum Generation Using Dispersion Engineered Near Zero Ultraflat Square-Lattice PCF around 1.55 *μ*m and Fabrication Tolerance Analysis

**DOI:** 10.1155/2014/276082

**Published:** 2014-12-31

**Authors:** Partha Sona Maji, Partha Roy Chaudhuri

**Affiliations:** Department of Physics and Meteorology, Indian Institute of Technology, Kharagpur 721302, India

## Abstract

In this work, a new design of ultraflat dispersion PCF based on square-lattice geometry with all uniform air holes towards broadband smooth SCG around the C-band of wavelength has been presented. The air hole of the inner ring was infiltrated with liquid of certain refractive indices. Numerical investigations establish a near zero ultraflattened dispersion of 0 ± 0.78 ps/nm/km in a wavelength range of 1496 nm to 2174 nm (678 nm bandwidth) covering most of the communications bands with the first zero dispersion wavelength around 1.54 *μ*m. With the optimized ultraflattened fiber, we have achieved a broadband SC spectrum with FWHM of 350 nm with the central wavelength of 1550 nm with less than a meter long of the fiber by using a picosecond pulse laser. We have also analyzed the sensitivity of the optimized dispersion design by small variations from the optimum value of the geometrical structural parameters. Our investigations establish that for a negative change of PCF parameters, the profile retains the smooth and flat SCG spectra; however, for a positive change, the smooth and a flat spectrum is lost. The new design of the fiber will be capable of covering huge diverse field of DWDM sources, spectroscopy, meteorology, optical coherence tomography, and optical sensing.

## 1. Introduction

Broadband supercontinuum generation (SCG) has been the target for researchers for its huge applications in the field of optical spectroscopy, dense wavelength division multiplexing (DWDM) sources, meteorology, optical coherence tomography, optical sensing, and so forth [1]. For broadband SCG, important aspects to be addressed are the spectral width and flatness over a broadband of wavelength. To generate a flat broadened SC, flat chromatic dispersion profile is one of the essential requirements. This requirement can be met by optimizing the design of the fiber and the pumping condition. Photonic crystal fibers (PCFs) [2, 3], which enjoy some excellent properties like wide band single-mode operation, great controllability over dispersion properties, and higher nonlinearity, can meet the demand for ultraflat dispersion in the communication wavelength. However, the dispersion slope of such PCFs cannot be tailored for wide wavelength range with air holes of the same diameter. Various complicated designs such as different core geometries [4–6] and multiple air-hole diameter in different rings [4, 5, 7–10] have been studied to achieve ultraflattened dispersion values over wider wavelength bandwidths. However, the design of the above-complicated structures of multiple air-hole diameters can be avoided in an alternative way. In this approach, the effect of variable air-hole diameter can be achieved by filling the air holes with liquids of certain refractive indices (RI). The infiltration of air hole has been demonstrated by filling the air holes with liquid crystals [11, 12] or by various liquids such as polymers [13], water [14], and ethanol [15]. Tunable photonic bandgap guiding (PBG) effect and long-period fiber grating have been successfully realized with liquid-filled PCFs [16]. There are certain issues related to the infiltration of liquid to the air holes: whether the fluid wets glass and how viscous it is which are discussed in detail in our previous work [17].

Out of the two regular transverse PCF geometries, square-lattice PCF has been found to be a better candidate compared to its triangular counterpart with respect to single-mode operation and broadband dispersion compensation. Square-lattice PCF is endlessly single-mode for higher *d*/Λ than triangular one [18] and it can better compensate the inline dispersion for broad dispersion compensation as it is having relatively nearer relative dispersion slope (RDS) with existing SMF28 [19]. With the equivalent parameters, square-lattice PCFs give higher values of effective area making it preferable for handling high power applications like SCG. Detailed numerical study of the square-lattice PCF for dispersion properties has already been done [19]. The practical feasibility of the square-lattice structure has been experimentally demonstrated with a study of the sonic bandgap based on square-lattice PCF perform [20].

In this paper, we have designed an ultraflat dispersion PCF based on square-lattice geometry with the inner air-hole ring infiltrated with a liquid of certain refractive indices around the C-band of wavelength with the first zero dispersion wavelength (ZDW) around 1550 nm. The numerical study shows that the proposed fibers can generate around 350 nm of flattened broadband SC generation in the IR wavelength ranging from 1375 nm to 1725 nm with less than a meter long of the fiber. The dependence on material dispersion of the infiltrating liquid towards the target dispersion has also been discussed. Fabrication tolerance of the optimized design has also been analyzed considering changes of the associated parameters by ±1%, ±2%, and ±3% from the optimized value.

## 2. Design of the Fiber and Analysis Method

**Figure 1:**
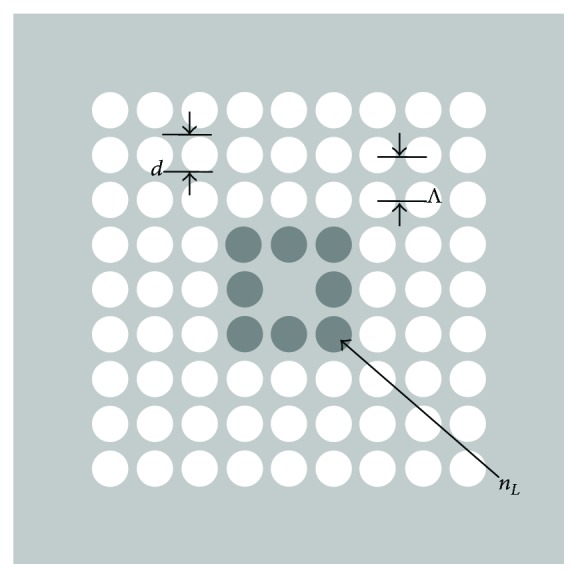
Cross section of the proposed photonic crystal fiber. The shaded regions represent air holes infiltrated with liquid with refractive indices *n*_*L*_.

The schematic of the designed fiber has been shown in Figure 1. It is well known to us that in usual triangular lattice PCF the conventional notations of air-hole diameter are “*d*” and hole-to-hole distance is “Λ.” In the analysis of the present square-lattice PCF structures, we use Λ as the hole-to-hole spacing in horizontal or vertical direction with *d* as the diameters of the air holes. Our designed PCF consists of four numbers of air-hole rings with the first air-hole ring infiltrated with liquid of certain refractive indices. Dispersion parameters as well as the modal field were calculated by using CUDOS MOF Utilities [21] that simulate PCF with multipole method [22, 23]. The numerical calculations are performed with MATLAB for the dispersion relation and nonlinear propagation of the pulse. The total dispersion (*D*) is computed with

(1) D=−λcd2Reneffdλ2.

Here *Re*[*n*_eff_] stands for the real part of the effective indices obtained from simulations and *c* is the velocity of light in vacuum.

## 3. Dispersion Analysis towards Ultraflat Dispersion PCF

**Figure 2:**
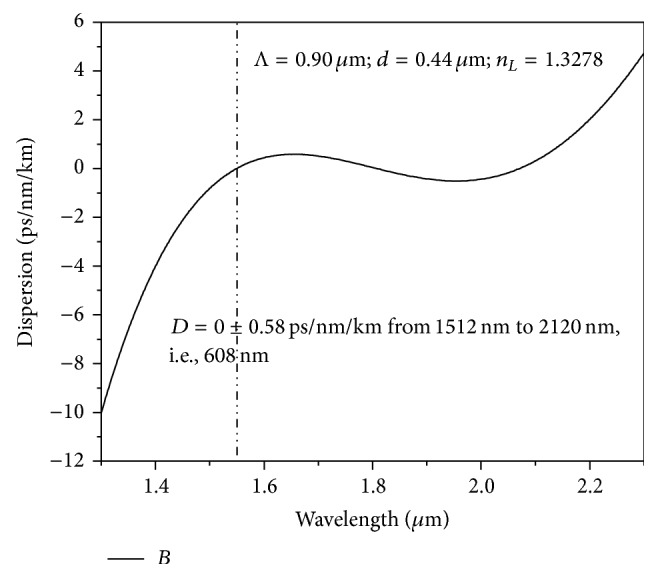
The ultraflat dispersion of 0 ± 0.58 ps/nm/km from 1512 nm to 2120 nm, that is, for a bandwidth of 608 nm obtained with Λ = 0.90 *μ*m and *d* = 0.44 *μ*m and *n*_*L*_ = 1.3278.

Our approach of optimization relies on varying multidimensional parameter space that consists of the liquid RI (*n*_*L*_), the pitch “Λ,” and air-hole diameter *d* to design ultraflat, near zero dispersion optical fibers. The dependence of the above parameters upon dispersion has been studied in our previous works [17, 24] which indicate that* varying the *Λ* influences the total dispersion*,* whereas d has the desired effect of modifying the dispersion slope*, and* varying n*_*L*_ *modifies both*. With the above conclusion, initially, we have designed one ultraflat near zero dispersion PCF with *D* = 0 ± 0.58 ps/nm/km based on square-lattice as shown in Figure 2 with Λ = 0.90 *μ*m, *d* = 0.44 *μ*m, and *n*_*L*_ = 1.3278. Here in this optimization we have considered wavelength independent liquid. The ZDW for this case comes out to be 1549 nm and the same has been indicated with the vertical dotted line in the figure. It should be mentioned that any material having RI of the above value can demonstrate the above dispersion nature. However, the material should be transparent for the considered wavelength window and thermally compatible with background silica. With a liquid of the above RI we need not require such a material (which is difficult to find) but to infiltrate once the PCF with air hole is fabricated. Next, we have considered one practical liquid (called as Liquid#1) available with M/s Cargille Lab, USA [25], whose RI is nearer to the above optimized value and which is given by Cauchy's equation (2). These liquids are basically refractive index matching liquids. The compositions are perfluorocarbon and chlorofluorocarbon (CFC) (not the types thought to affect the ozone). The liquids have a broad range of applications in diverse fields like identification, mounting media, optical analysis, refractometry, spectrometry, strain analysis optical coupling, optical lenses, electrooptics, fluid flow, and so forth. A detail of the applications can be found from the manufacturer website [25].

Cauchy equation of the oil is as follows:

(2) $$\begin{array}{c} \text{Liquid}\#1\text{:}\;\;n1\left(\lambda \right)=1.3289114+\frac{210577}{\lambda^{2}}+\frac{3.006168\times 10^{10}}{\lambda^{4}}, \end{array}$$

where *λ* are in Angstrom.

**Figure 3:**
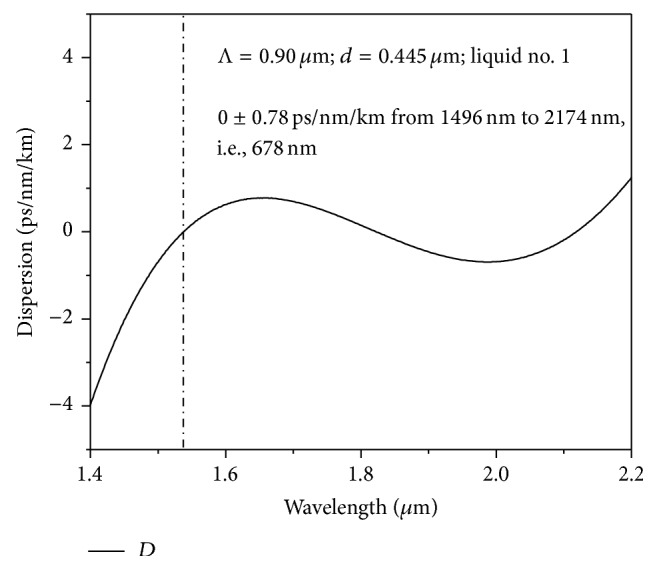
The ultraflat dispersion of 0 ± 0.78 ps/nm/km over 1496 nm to 2174 nm, that is, for a bandwidth of 678 nm obtained with Liquid#1 with Λ = 0.90 *μ*m and *d* = 0.445 *μ*m.

**Figure 4:**
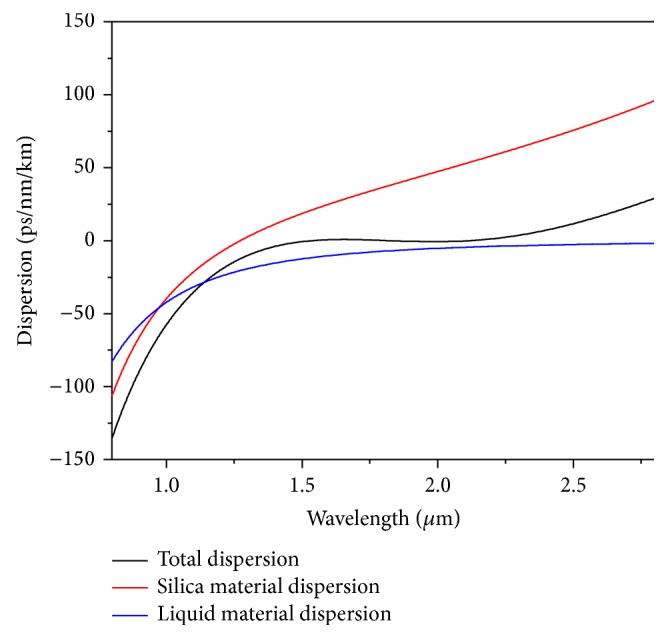
Contribution of the material dispersion of Liquid#1 along with the background silica material dispersion towards the total ultraflat dispersion for the fiber with Λ = 0.90 *μ*m and *d* = 0.445 *μ*m. Material dispersion of the liquid contributes significantly towards achieving ultraflat near zero dispersion.

**Figure 5:**
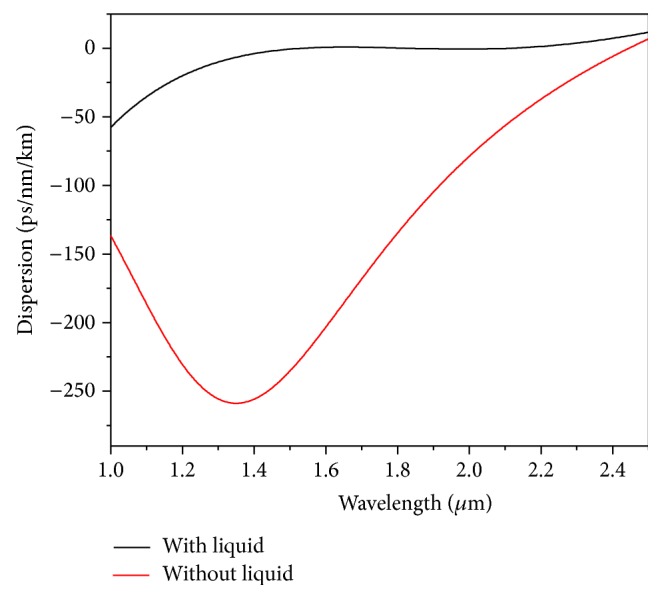
The effect of liquid infiltration in the first air-hole ring upon dispersion. Not only has the dispersion values, the slope of the graph been drastically altered.

With this liquid, we adjust the other structural parameters to achieve an ultraflattened dispersion of *D* = 0 ± 0.78 ps/nm/km as shown in Figure 3 with Λ = 0.90 *μ*m, *d* = 0.445 *μ*m. The ZDW corresponding to the above case comes out to be 1540 nm and the same has been shown in the above figure with a vertical dotted line. The value is significant as the pumping condition depends upon the ZDW [1]. The dependence of the liquid material dispersion upon total dispersion has been presented in Figure 4. The figure clearly reveals a significant contribution of the liquid towards ultraflat dispersion.  Figure 5 shows a comparative study giving the dispersion properties with and without the liquid with the optimized PCF design. The figure clearly establishes the claim of Figure 4 of great influence by the liquid towards dispersion engineering. Our numerical investigation establishes that mode field is almost confined inside the core region. So the investigated properties of broadband SCG around 1550 nm will depend upon the core material only. Subsequently the only effects the liquids will have will be the RI modification.

The selective hole-filling technique provides a couple of advantages.* Firstly*, all the air holes have the same diameter, which is easier to be fabricated compared to fibers with multiple different submicron air-hole sizes.* Secondly*, a regular PCF with selective infiltration with a liquid provides huge flexibility for tremendous applications like birefringence, parametric amplifier, and so forth.

There are certain issues related to the infiltration of liquid to the air holes: whether the fluid wets glass and how viscous it is. If the liquid does not wet glass then surface tension will oppose entry of the liquid into the hole, making it difficult to fill. One can work out the pressure needed to push such a liquid into a hole given its surface tension and contact angle, and it is likely to require a pressure greater than 1 atmosphere for a 0.40 *μ*m air hole. In that case, a vacuum pump would be insufficient. If the fluid does wet glass then the hole should fill but the fill speed will depend on viscosity. It can be worked out how quickly it will fill using the expressions for Poiseuille flow in a pipe. In other words, the holes can be filled (and how quickly), with the given values for surface tension, contact angle, and viscosity. With the technology advancing very fast submicron filling of air holes will not be very difficult to achieve.

## 4. Supercontinuum Generation (SCG) with the Ultraflat Dispersion PCF

Supercontinuum generation (SCG) is a nonlinear process that depends on the nonlinear response and dispersion characteristics of the fiber [1, 26]. For a flat broadband SCG, a near zero flat dispersion profile is required. The pulse propagation for SCG through the fiber has been calculated using nonlinear Schrödinger equation (NLSE) [26] as given in

(3) ∂A∂Z+α2A−∑n≥2in+1n!βn∂nA∂Tn =iγ1−fRA2A−iw0∂∂TA2A  +iγfR1+iw0A∫0∞hRτAz,T−τ2∂τ,

where *A* is the complex amplitude of the optical field, *α* is the attenuation constant of the fiber, *β*_*n*_ (*n* = 2 to 10) is the *n*th order of the Taylor series expansion of the propagation constant around the carrier frequency, *γ* is the nonlinear coefficient with *ω*_0_, the input pulse frequency, and *f*_*R*_ is the fractional contribution due to delayed Raman function *h*_*R*_(*τ*).

**Figure 6:**
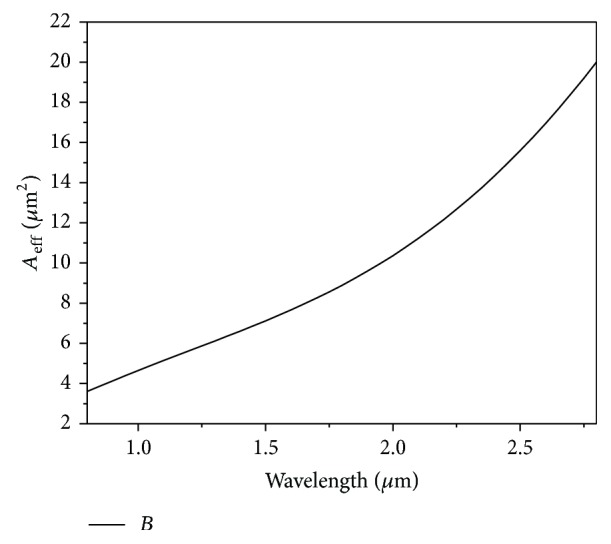
Effective area variation for ultraflat dispersion PCF with Λ = 0.90 *μ*m, *d* = 0.445 *μ*m with Liquid#1.

SCG has been numerically calculated by solving the NLSE with the split step Fourier step based beam propagation code developed by COSTP11 [27]. The nonlinear parameter *γ* has been calculated using

(4) $$\begin{array}{c} \gamma =\frac{2\pi n_{2}}{\lambda A_{\text{eff}}}, \end{array}$$

where *n*_2_ is the nonlinear refractive indices of the material and *A*_eff_ of the effective area of the fiber at the pumping wavelength. The effective area variation of the proposed fiber has been shown in Figure 6. As can be observed from the figure that, with the increase of the operating wavelength, the effective area increases signifying the spreading of the pulse for higher wavelength. The nonlinear parameter was found to be 15.31 w^−1^·km^−1^ at the pumping wavelength of 1550 nm.

**Figure 7:**
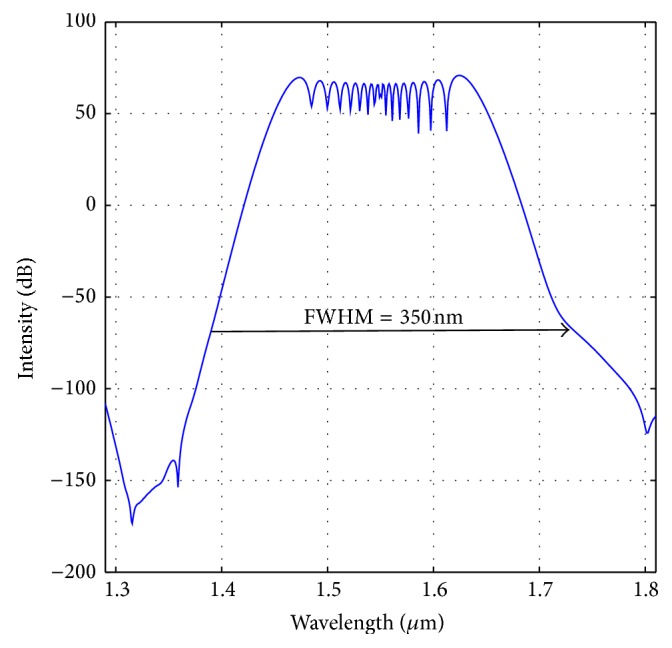
Optical spectrum of the optimized PCF with Λ = 0.90 *μ*m, *d* = 0.445 *μ*m with Liquid#1 after travelling a distance of 0.9 m.

**Figure 8:**
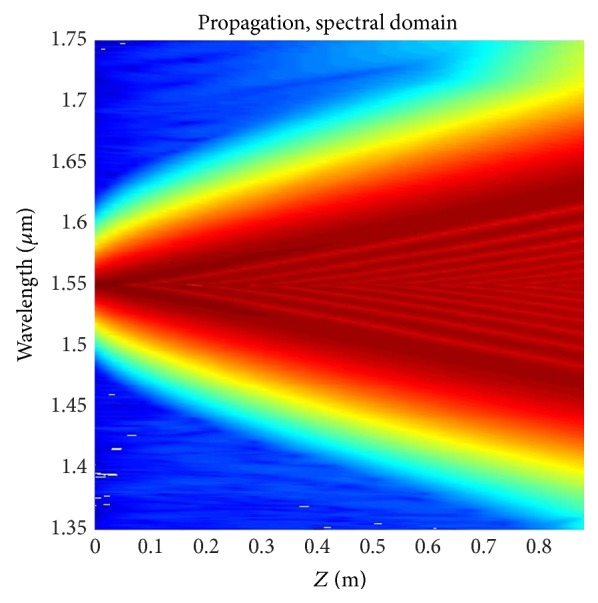
Spectral evolution as a function of length of the fiber for the optimized PCF with Λ = 0.90 *μ*m, *d* = 0.445 *μ*m with Liquid#1.

For numerically solving the SCG for the designed ultraflat PCF we have considered sech^2^ pulse as the input with a value of full width at half maximum (FWHM) of 1.0 ps. The power of the input pulse has been fixed at 4.5 kW with the center/pumping wavelength of 1550 nm. A possible source for such a pump can be the commercially available fiber laser emitting around 1550 nm of wavelength [28]. The calculated values of *β*_2_, *β*_3_, *β*_4_, *β*_5_, *β*_6_, *β*_7_, *β*_8_, *β*_9_, and *β*_10_ are −0.20852 ps^2^/km, 0.02129 ps^3^/km, 2.24055*e* − 4 ps^4^/km, −8.5368*e* − 7 ps^5^/km, −3.45006*e* − 9 ps^6^/km, 1.25875 *e* − 10 ps^7^/km, −1.84629 *e* − 12 ps^8^/km, 1.3465*e* − 14 ps^9^/km, and −1.95697*e* − 17 ps^10^/km, respectively. The output spectrum of the optimized fiber after travelling distance of 0.9 meter has been shown in Figure 7. The spectrum calculation has been performed according to Begum et al. [29]. Numerical calculation reveals a FWHM of 350 nm with the central wavelength of 1.55 *μ*m after travelling only a distance of 0.9 meters. The evolution of the pulse as it travels through the fiber has been demonstrated in Figure 8.

## 5. Fabrication Tolerance

**Figure 9:**
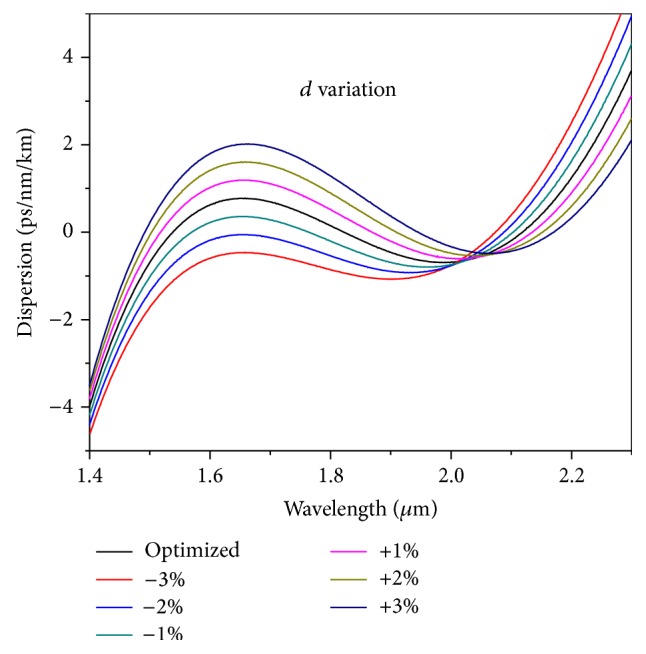
Dependence of dispersion values on varying air-hole diameter (*d*) for fixed values of hole-to-hole spacing (Λ) and infiltrated Liquid#1.

Here in this section we discuss the fabrication tolerance of the optimized design. For this analysis, we have presented the effect of change of the air-hole diameter “*d*” and hole-to-hole distance “Λ” from the optimized values by changing ±1%, ±2%, and ±3%, respectively. We have not considered the variation of RI of the liquid as the liquid RI is constant for a particular liquid.  Figure 9 presents the effect of change of “*d*” from the optimized value. For an increment of the air-hole diameter the oscillation of the dispersion increases and the flatness decreases and the dispersion nature does not retain the required ultraflat nature. For +1%, +2%, and +3% change of the diameter, the oscillation increases to 0 ± 1.19 ps/nm/km, 0 ± 1.61 ps/nm/km, and 0 ± 2.02 ps/nm/km, respectively. So on an average for an increment of each percentage of the air-hole diameter the oscillation changes by 0.41 ps/nm/km. For a decrease of “*d*” the oscillation decreases; however, the value of the first ZDW, which is an important parameter for pumping condition, becomes 1570 nm for −1% from the optimized value. However, for other values −2% and −3% of the dispersion nature become all normal type; that is, dispersion values are all negative. To have broader SC spectra we need the dispersion to have one ZDW where the pumping wavelength should be introduced. However, in the last two cases (−2% and −3% from the optimized one) the condition is nullified. However, these cases can be applied for all normal broadband condition as has been investigated in a different research recently [30]. The all normal dispersion can be optimized closest to zero dispersion value for a maximum bandwidth. With the all normal near zero dispersion nature the soliton effect which contributes to asymmetric and rough spectrum around the pumping wavelength can be reduced and we can achieve wideband smooth SCG spectrum. So, both competing designs (all normal near zero and near zero ultraflat dispersion) will provide broadband SCG for a sufficient wavelength window. But for the present study they do not remain useful anymore.

**Figure 10:**
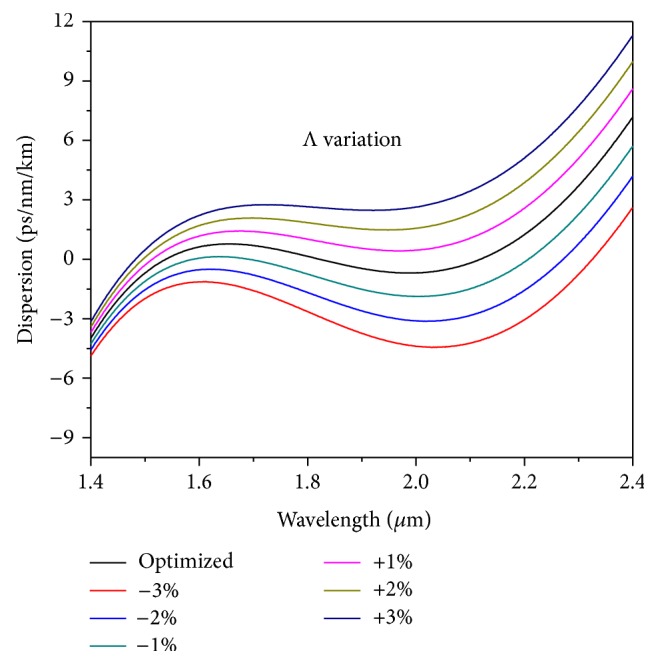
Dependence of dispersion values on varying hole-to-hole spacing (Λ) for fixed values of air-hole diameter (*d*) and infiltrated Liquid#1.

The effect of change of Λ from the optimized value has been presented in Figure 10 for ±1%, ±2%, and ±3% values. The figure clearly reveals that change of Λ mostly changes the values of the dispersion without much change in the slope. With an increase of the Λ values, the oscillation decreases and most importantly the value of ZDW gets blue shifted. In the above cases, the oscillations do not remain oscillating about the zero dispersion value; rather they have only one ZDW throughout the wavelength range. For change of Λ by +1%, +2% and + 3% than the optimized value, the peak dispersion value reaches to 1.42 ps/nm/km, 2.08 ps/nm/km and 2.75 ps/nm/km, respectively. So a change of dispersion value on an average of 0.66 ps/nm/km occurs for each percentage change of Λ compared to the optimized value. For −1% change of the optimized value the oscillation increases, though a value of ZDW still could be observable, whereas the same is absent for −2% and −3% change compared to the optimized value. In the last two cases, the dispersion becomes all normal dispersion type with no ZDW. These structures are not going to be of any help for our present target. However, with proper adjustment of the parameters they can be applied for other applications like all normal broadband sources and so forth. The above discussion suggests that for a change of the optimized parameters (both *d* and Λ) up to ±1% the optimized design still retains the ultraflat nature through the oscillation increases through pumping condition changes. It should be noted that this analysis of percentage change of optimized parameters serves to provide an upper limit on the rigorousness of the fabrication imperfections, since in reality not all the air holes are distorted from their optimum value or location in the same way, and some averaging effect is likely to occur.

## 6. Effect of Percentage Change upon Broadband SCG

**Figure 11:**
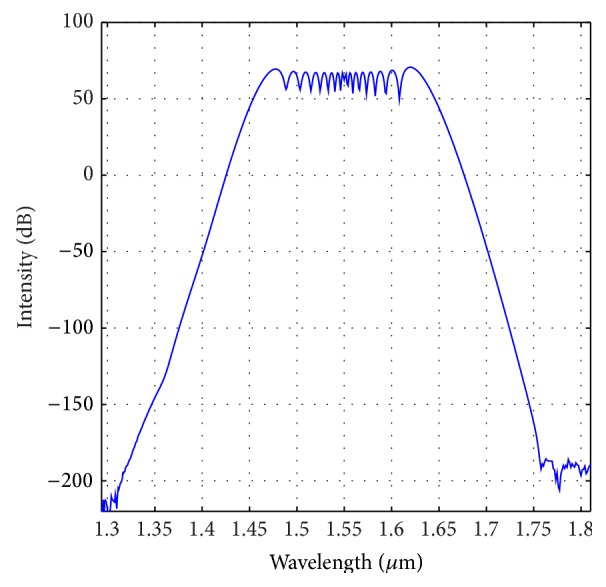
Optical spectrum for −3% change of “*d*” to the optimized PCF as shown in Figure 3.

**Figure 12:**
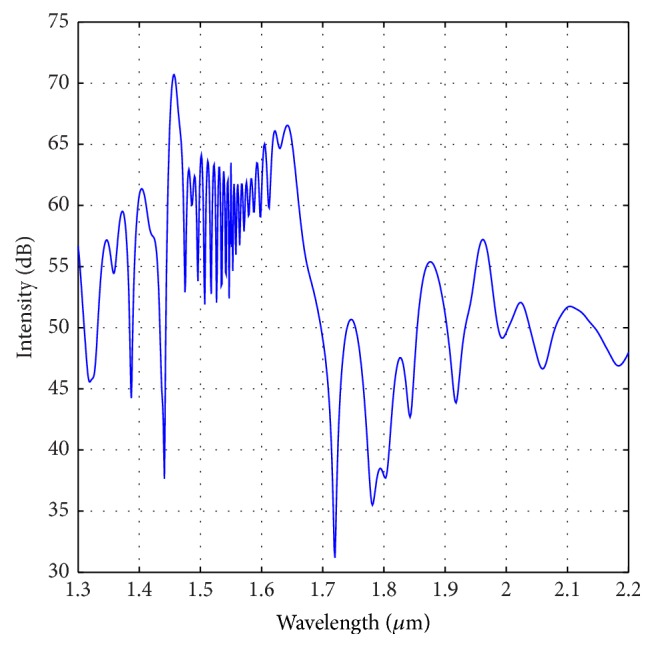
Optical spectrum for +3% change of “*d*” to the optimized PCF as shown in Figure 3.

In this section, we investigate the SCG property when the optimized parameters are changed by a certain amount. For this case, we have considered ±3% change of the optimized parameters as mentioned in the previous section. To begin with, we have considered the effect of percentage change of “*d*.” Figure 11 shows the SCG for the dispersion profile of −3% compared to the optimized one. We can see that with this profile we could generate broadband SCG similar to the nature observed in Figure 7. However, the FWHM of this spectrum is reduced to around 310 nm. Again, if we consider the dispersion profile for +3% changes from the optimized dispersion shown in Figure 9, we can see that (from Figure 12) the obtained SCG does not retain smooth nature as shown in Figure 12. Rather the spectrum spreads to wider wavelength without maintaining symmetric and smooth nature.

**Figure 13:**
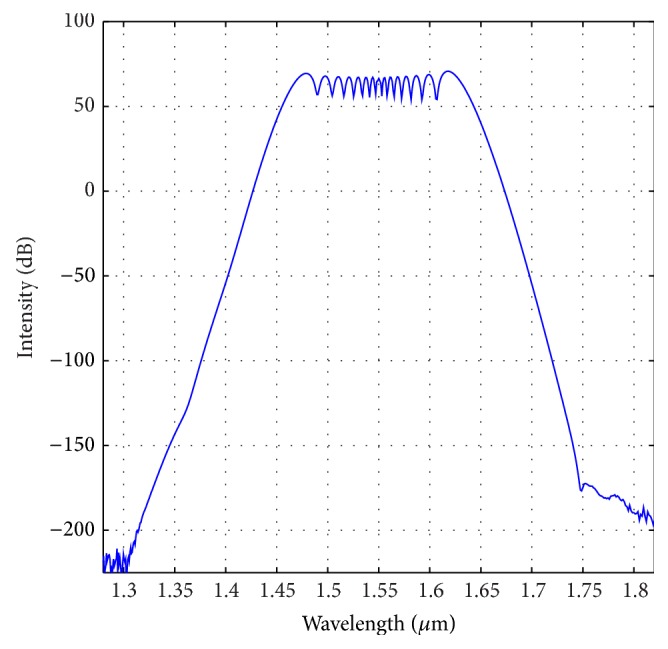
Optical spectrum for −3% change of “Λ” to the optimized PCF as shown in Figure 3.

**Figure 14:**
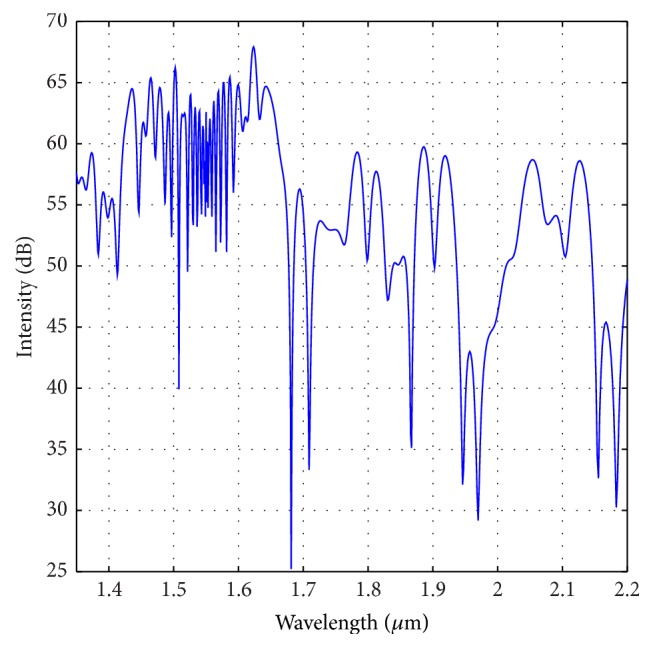
Optical spectrum for +3% change of “Λ” to the optimized PCF as shown in Figure 3.

We have also investigated the change of SCG property for the percentage change of “Λ” as mentioned above with an amount of ±3% than the optimized value. For the change of Λ by −3%, we could achieve a broadband SCG as shown in Figure 13 with reduced FWHM of 315 nm. For a positive change of Λ by the same amount (+3%), the spectrum is not smooth and flat as shown in Figure 14.

The above nature can be explained on the basis of the dispersion profile. For the negative change of the PCF parameters, the dispersion profile does retain the all normal profile and soliton fission which is responsible for breaking of pulses is absent for this case. For the case of positive change (+3%), the dispersion profile is not all normal one and subsequently soliton fission comes into play and as a result, we have wideband SCG spectrum without the smooth and flat spectrum at the output.

## 7. Conclusion

We have demonstrated a new design of near zero ultraflat dispersion based on square-lattice PCF with the inner air-hole ring infiltrated with liquid of certain RI. The design has been further extended for a broadband SCG with a picosecond (ps) pulse laser around the C-band of wavelength. Our numerical analysis establishes an ultraflat near zero dispersion of *D* = 0 ± 0.78 ps/nm/km from 1496 nm to 2174 *μ*m, that is, for a bandwidth of 678 nm. With the above designed PCF, we have obtained a flat, smooth, and broadband spectra ranging from 1350 nm to 1725 nm, that is, for a bandwidth of 375 nm with only a meter long of the fiber. Fabrication tolerance of the optimized design has been discussed and it was found out that a percentage change of ±1 compared to the optimized value still keeps the ultraflat dispersion nature intact. We have also shown that, for a negative percentage change of both air-hole diameter and hole-to-hole spacing, the SCG retains the smooth and flat spectra however with reduced FWHM. On the other hand, for a positive change of the parameters, the SCG does not retain the smooth and flat nature. The significance of this work is that it provides a new type dispersion engineered silica based PCF for near IR SC source with flat shape, broadband properties with about a meter-long fiber which can be useful for applications in metrology, spectroscopy, and optical coherence tomography in the near-infrared region.
